# Interspecies hybridization between *Auricularia cornea* cv. Yu Muer and *Auricularia heimuer* cv. Bai Muer through protoplast fusion

**DOI:** 10.3389/fmicb.2023.1280420

**Published:** 2023-10-30

**Authors:** Keqing Qian, Zhengxiang Qi, Anran Xu, Xiao Li, Bo Zhang, Yu Li

**Affiliations:** ^1^Northeast Asian Specialty Germplasm Resources Innovation Centre, Jilin Agricultural University, Changchun, China; ^2^Engineering Research Center of Chinese Ministry of Education for Edible and Medicinal Fungi, Jilin Agricultural University, Changchun, China; ^3^Joint International Research Laboratory of Modern Agricultural Technology, Ministry of Education, Jilin Agricultural University, Changchun, China

**Keywords:** edible mushrooms, color variations, interspecies hybridization, protoplast inactivation, *Auricularia*

## Abstract

Color variations in cultivated edible mushrooms present novel and potentially valuable alternatives to the research and cultivation industries. We collected, identified, and domesticated a white strain of *Auricularia cornea* and a white strain of *Auricularia heimuer* from China. However, due to an unstable phenotype and stricter requirements on environment and management technology, the production and utilization of *Auricularia heimuer* cv. Bai Muer make slow progress. Outcrossing is an essential means to broaden the intraspecific genetic resources to expand the gene pool and compensate for the limitations of related species hybridization. In this study, interspecies hybridization between *Auricularia cornea* cv. Yu Muer and *Auricularia heimuer* cv. Bai Muer was conducted using polyethylene glycol (PEG)-induced double-inactivated protoplast fusion. Apart from the functional complementation of double-inactivated protoplasts, the hybrids were characterized by colony morphology, antagonistic test, primordial morphology, and polymerase chain reaction (PCR) fingerprinting. The results suggested that the hybrids and their parents showed significant differences in their colony morphology. Moreover, positive barrage reactions were observed between each parent and hybrid. Inter-simple sequence repeat (ISSR) and start codon targeted (SCoT) profile analysis of fusants and parents depicted that fusants contained polymorphic bands, which indicated the rearrangement and deletion of deoxyribonucleic acid (DNA) in the fusants. Yellowish-white primordia were obtained from two hybrids. Protoplast fusion may reinforce the genetic potential and provide an ideal alternative for breeding albino *Auricularia*.

## 1. Introduction

*Auricularia* Bull. (family Auriculariaceae, order Auriculariales) is an important wood-decaying fungal genus widely distributed worldwide (Wu et al., 2014). Moreover, it is one of the earliest cultivated mushrooms in the world and was first recorded in Tang Materia Medica, written by Gong Su (Yuan et al., 2019). It has traditionally been consumed as food and medicine for over 1,000 years in China (Miao et al., 2020). As the primary producer of cultivated *Auricularia* in the world, China’s output reached approximately 9.24 million tons in 2021, representing over 90% of the global production (China Edible Fungi Association, 2022). *Auricularia cornea* Ehrenb. and *Auricularia heimuer* F. Wu, B.K. Cui, and Y.C. Dai are the main species commercially cultivated in China.

Edible mushrooms with color variation have high research value and commercial value, such as Agaricus bisporus (J.E. Lange) Imbach, Flammulina filiformis (Z.W. Ge, X.B. Liu & Zhu L. Yang) P.M. Wang, Y.C. Dai, E. Horak & Zhu L. Yang, Hypsizygus marmoreus (Peck) H.E. Bigelow (Lee et al., 2008; Liu et al., 2013, 2016). *Auricularia cornea* cv. Yu Muer is an albino mutant strain of *A. cornea* with numerous biological activities, such as antidiabetic, antinephritic, antioxidant, anticoagulant, and hepatoprotective effects (Wang et al., 2019; Li et al., 2021). The pigment in the fruiting body of *A. cornea* was γ-glutaminyl-3,4-dihydroxy-benzoate. In the process of synthesizing pigment, the key enzymes were polyphenol oxidase and 20 other enzyme genes (Ma et al., 2023). In addition to its white color, the popularity of this mushroom is due to its high nutritional content and short production cycle (45–55 days). The mushroom is grown on an industrial scale in many regions of China because it has a high-yielding capacity and low output cost, is adaptable to a different environment, and is resistant to many pathogens (Chen et al., 2021).

In 2019, the white variety of *A. heimuer* was successfully domesticated and cultivated at Jilin Agricultural University (Li et al., 2019). According to the latest statistics, *A. heimuer* (ranks second in production) is more popular than *A. cornea* (ranks seventh in production) in China due to its flavor, slippery texture, and unique taste. However, *A. heimuer* cv. Bai Muer has a longer production cycle and higher output cost than *A. cornea* cv. Yu Muer, so it needs stricter requirements on environment and management technology. In addition, the color of the fruiting body is easily affected by light during the cultivation period. Therefore, selecting new strains of *Auricularia* with good characteristics is of great importance.

There are many ways to breed new strains, for example, artificial selection breeding, cross-breeding, protoplast fusion breeding, mutation breeding, and genetic engineering breeding. A lot of traditional mushroom breeding methods have been carried out intraspecifically. However, due to a lack of basic knowledge of the genetics and breeding system of this crop, advances in research on mushroom breeding and production are very limited compared with other crops. Moreover, the fruiting body of a mushroom is a complex organism with a series of complex characteristics. Many of these characteristics, especially those related to yield, are controlled by multiple genes (Chakravarty, 2011).

Gene transfer using protoplast fusion is a non-conventional method that is used to break down the natural barrier to gene exchange encountered in conventional breeding systems. Protoplast fusion technology can be performed intraspecifically, interspecifically, intergenerically, and even inter-hetero-generically (Dhitaphichit and Pornsuriya, 2005). The course of biological processes can be significantly influenced by protoplast fusion between different species. Through this process, gene control can be deregulated either positively or negatively, and metabolic pathways may be combined to create new metabolites. This can result in high yields, fast spawn runs, tolerance to adverse conditions, utilization of various agricultural waste, unique taste, attractive color, enhanced nutritive value, and medicinal properties in mushrooms (Selvakumar et al., 2015; Raman et al., 2021). Hybrids constructed by protoplast fusion in several mushrooms have been reported (Mallick and Sikdar, 2014). Interfamily hybrid strains with high biological efficiency and cold-tolerant ability have been obtained through protoplast fusion (He et al., 2018). A successful interspecific protoplast fusion has been carried out between the two edible mushroom strains *Lentinula edodes* (Berk.) Pegler and *Coriolus versicolor* (L.) Quél. (Kim et al., 1997). Somatic hybrids between *Calocybe indica* Purkay & A. Chandra and *Pleurotus fiorida* Singer showed a significant increase in bio-eficiency and γ-linoleic acid content (Chakraborty and Sikdar, 2010). The structural investigation of polysaccharides obtained from somatic hybrid mushrooms through protoplast fusion showed that they are different from the polysaccharides isolated from the fruit bodies of parental strains and exhibited strong immune activation of macrophages, splenocytes, and thymocytes (Patra et al., 2011; Maity et al., 2013; Maji et al., 2013; Sen et al., 2013). Therefore, distant hybridization can introduce important quantitative and qualitative traits, such as high bio-efficiency, good palatability, and a shorter cropping period, from either of the parents into their progeny. Interspecies hybridization between white *Auricularia* through protoplast fusion can enhance genetic potential and offer an excellent alternative for breeding edible mushrooms.

Thus, considering the beneficial characteristics of the two parents, the present study carried out the protoplast fusion between *A. heimuer* cv. Bai Muer and *A. cornea* cv. Yu Muer to obtain new intergeneric strains of albino *Auricularia* with improved characteristics. In our study, we successfully developed 10 hybrids, which were successfully characterized by microstructure, mycelial morphology, inter-simple sequence repeat (ISSR), and start codon targeted (SCoT) analysis.

## 2. Materials and methods

### 2.1. Strains and media

The *A. cornea* cv. Yu Muer strain (MC6), the *A. heimuer* cv. Bai Muer strain (JAUH-W-591), monokaryotic strains of *A. cornea* cv. Yu Muer (D-MC6), and *A. heimuer* cv. Bai Muer (D-JAUH-W-591) were preserved at Jilin Agricultural University (Changchun, China). Vegetative cultures of both strains were maintained on potato dextrose agar (PDA) medium, containing 20 g/L of glucose, 2 g/L of KH_2_PO_4_, 2 g/L of MgSO_4_·7H_2_O, 1.5 g/L of agar, and 1 L of potato juice (He et al., 2018). Before protoplast isolation, the strains were grown in liquid malt yeast extract glucose (MYG) medium (10 g/L of malt, 4 g/L of yeast extract, and 10 g/L of glucose, pH = 6.2) under stationary conditions for 10 days at 30°C (Chakraborty and Sikdar, 2008; Xu et al., 2012). The same MYG medium supplemented with 0.6 M MgSO_4_ and 2% agar was used as a regeneration medium.

### 2.2. Isolation of protoplasts

Monokaryotic mycelia derived from a single spore isolate of each species were incubated for 10 days at 28°C in 100 mL of liquid MYG medium for static culture. Cultures were harvested by the filter (0.22 μm), washed twice with distilled water, and dried with sterile paper. Then, 200–300 mg of mycelium was added to a 1 mL aliquot of lywallzyme solution (2%, purchased from the Guangdong Institute of Microbiology), which contained 0.6 M osmotic stabilizer and was incubated at 30°C for 7 h. The suspension was filtered and centrifuged at 3000 × g for 5 min. The obtained protoplasts were collected and washed twice with a 0.6 M osmotic stabilizer. The total yield was calculated using a hemocytometer (Wang et al., 2017). Finally, purified protoplast pellets were suspended in 200 μL of osmotic stabilizer solution for further use.

### 2.3. Inactivation of protoplasts

The protoplast suspensions of *A. heimuer* cv. Bai Muer and *A. cornea* cv. Yu Muer were inactivated by heat and ultraviolet (UV) radiation, respectively. For heat inactivation, the protoplasts were treated at 55, 60, and 65°C for 10, 20, and 30 min, respectively; for UV inactivation, protoplasts were placed 30 cm away under a 15 W UV lamp for 1, 3, 5, 8, and 10 min. After serial dilution, the inactivated protoplasts were plated on the regeneration MYG medium to check the inactivation effect (He et al., 2018). The medium was cultured at 28°C, and the number of regenerated colonies was recorded after 15 days. Protoplasts without inactivation were set as the control group. The inactivated protoplasts were then used for fusion (Zhao et al., 2011).

### 2.4. Fusion of protoplasts

An equivalent amount of inactive protoplasts of *A. heimuer* cv. Bai Muer and *A. cornea* cv. Yu Muer was mixed in a test tube and centrifuged at 1,000 × g for 5 min. The supernatant was rinsed off, and 1 mL of sterilized polyethylene glycol (PEG 4000; 30 g PEG in 100 mL 0.05 M CaCl_2_·2H_2_O) was added to the protoplasts in the test tube and incubated at room temperature for 30 min (Moturi and Charya, 2009). During this period, protoplast fusion was followed by observation under the optical microscope (Nikon, Japan). The fused protoplasts were centrifuged at 1,000 × g for 5 min. The supernatant was rinsed off, and protoplasts were washed twice with the osmotic stabilizer and added 1 mL of osmotic stabilizer again. They were then serially diluted, and approximately 0.1 mL from the protoplast suspension was coated in MYG with 0.6 M MgSO_4_ at 25°C until colonies developed (Zhao and Chang, 1996). Protoplasts from the same parent strains were also fused as controls. Only the progeny that continued growing on the regeneration medium were considered fusion hybrids. The nuclear phase of the putative hybrid stained with 4′,6-diamidino-2-phenylindole (DAPI) dye was observed by fluorescence microscopy. These procedures excluded the possibility of a dual culture.

### 2.5. Identification of hybrids

#### 2.5.1. Antagonistic reactions

Hybrid mycelia, on slabs of PDA, were inoculated at a distance of 2 cm, with three in each Petri plate (i.e., the two parent cultures and a single hybrid). The plates were incubated at 25°C for 14 days, after which the point of contact zone was observed.

#### 2.5.2. Spawning and fruiting test

All hybrids were subjected to a fruiting test in the laboratory. The spawn substrate, which consisted of (w/w) 40% flake hardwood sawdust (4 mm × 6 mm), 37.5% powdered hardwood sawdust, 11% bran, 10% corncob, 1% gypsum, 0.5% lime, pH = 7, and 58–60% water, was autoclaved at 121°C for 120 min. After spawning, when the mycelia showed complete colonization in the substrate, several “V” pores were made all over the surface of the polypropylene packet (approximately 2 cm apart). The temperature was then maintained at 22–28°C, and the relative humidity was adjusted to 85–90%. After pin head emergence through the pores on the polypropylene packets, high humidity was maintained by misting the room. Ventilation and light were required for healthy fruiting body development. If a strain did not form any primordia in all triplicate bags after 25 days, it was considered sterile. The morphology of the fruiting bodies of the hybrids was compared with that of the parents.

#### 2.5.3. Inter-simple sequence repeat and start codon targeted analysis

Genomic deoxyribonucleic acid (DNA) was isolated from actively growing mycelia using a DNA Extraction Kit (Beijing CoWin Biotech Co., Ltd.). The ISSR primers used in the test are shown in Table 1. The ISSR amplification condition was as follows: 5 min initial denaturation at 94°C; 60 s initial denaturation at 94°C; 35 cycles consisting of 45 s denaturation at 52–58°C; 1 min extension at 72°C; and a final extension for 10 min at 72°C. Reaction termination was conducted at 4°C. ISSR-PCR reaction system (20 μL) was as follows: 10 μL of PCR Master Mix (2 X), 7.5 μL of dd H_2_O, 1 μL of ISSR primer, and 1.5 μL of DNA. The final PCR products were separated by electrophoresis on a 1.0% (w/v) agarose gel (Chiu et al., 1995). According to Zhao et al. (2013), SCoT amplifications were performed with modifications. The SCoT primers used in the test are shown in Table 2.

**Table 1:** Sequence and Annealing temperature of nine inter-simple sequence repeat (ISSR) primers.

Primer	Sequence	Annealing temperature (°C)
3	5′ (CA)8G 3’	55.5
4	5′ (ATG)6 3’	56
6	5’CCG ACTCGA GNN NNN NATGTGG 3’	57.5
p2	5’ BDB(ACA)5 3’	55
p4	5′ (CAC)4 SC 3’	58
p11	5′ (AC)8\u00B0C 3’	56
p13	5′ (GA)8 YG 3’	57
p14	5′ (AG)8 YT 3’	56
p15	5′ (AG)8 YC 3’	57.5

Note: B = (C,G,T); D = (A, G, T); S = (G, C); Y = (C, T); N = (A, T, C, G).

## 3. Results

### 3.1. Preparation and regeneration of protoplasts

**Figure 1:**
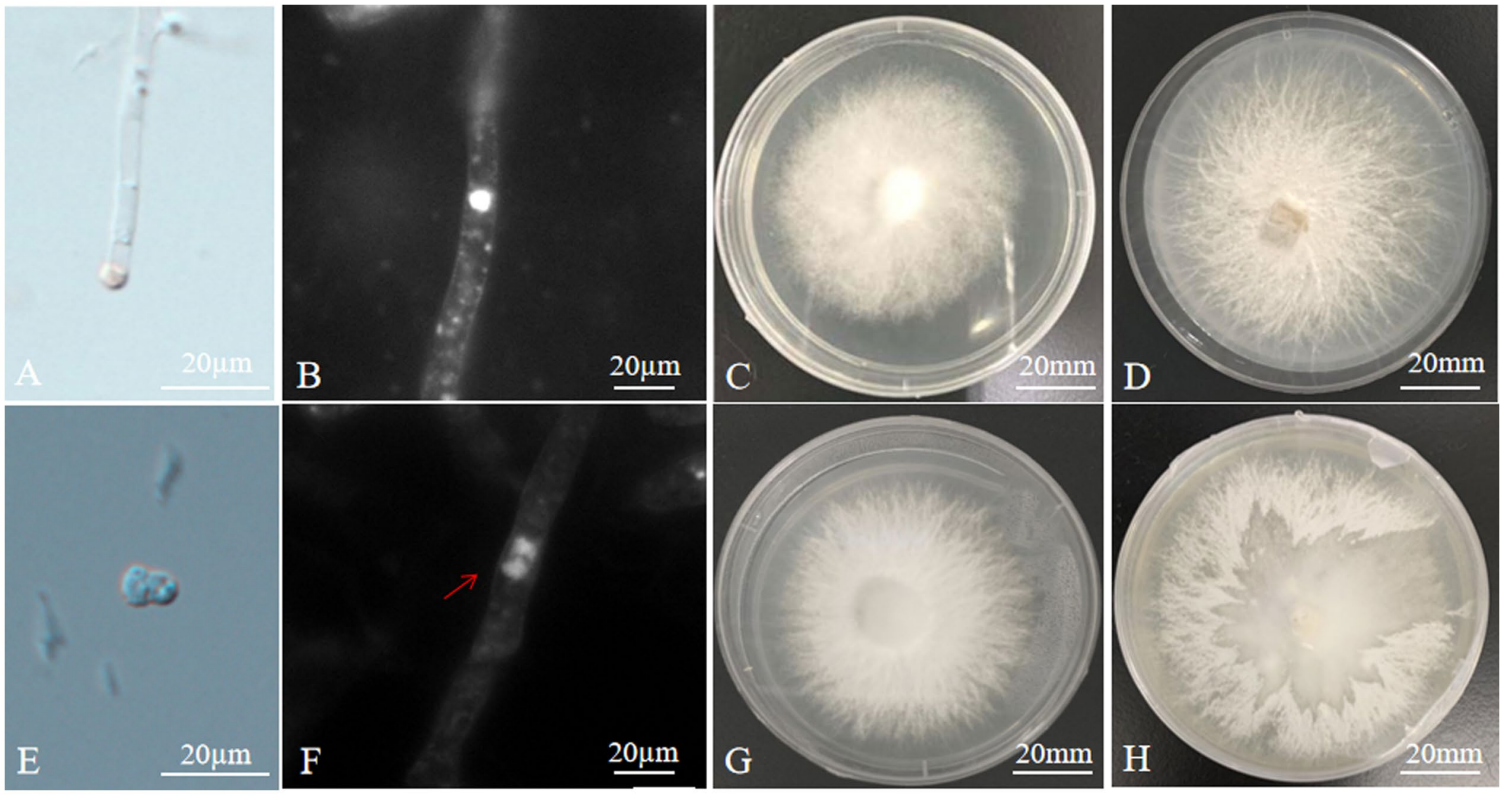
Microstructure and mycelial morphology of parents and hybrids **(A)** isolation of protoplast; **(B)** monokaryotic mycelium of *A. heimuer* cv. Bai Muer; **(C)** colony morphology of *A. heimuer* cv. Bai Muer; **(D)** colony morphology of *A. cornea* cv. Yu Muer; **(E)** protoplast fusion; **(F)** dikaryotic mycelium of hybrid; **(G)** colony morphology of hybrid strains R2; and **(H)** separation of one parent from unstable heterokaryons.

Sufficient protoplasts could be obtained by using mycelium aged 10 days under the conditions of an enzymatic hydrolysis temperature of 30°C, an enzymatic hydrolysis solution concentration of 2.0%, and an enzymatic hydrolysis time of 7 h. The protoplast yield was 7.84 × 10^7^ CFU/mL in *A. heimuer* cv. Bai Muer and 7.36 × 10^7^ CFU/mL in *A. cornea* cv. Yu Muer. The regeneration percentage of *A. heimuer* cv. Bai Muer protoplast was found to be 6.6%, while the rate of *A. cornea* cv. Yu Muer was 6.4%. The process of protoplasts being released from young hyphae was observed under a microscope (Figure 1A). Protoplast regeneration was observed in MYG medium containing 0.6 M MgSO_4_ after 4 days.

### 3.2. Heat and ultraviolet inactivation of protoplasts

Inactivation of *A. heimuer* CV. Bai Muer protoplasts at 60°C for 20 min yielded good results, as no protoplasts regenerated in the MYG medium with 0.6 M MgSO_4_ after this treatment. After 3 min of UV inactivation, the inactivation rate of the *A. cornea* CV. Yu Muer protoplast was 100%, and the regeneration rate was 0.

### 3.3. Fusion and regeneration of protoplasts

The contact and fusion of protoplasts induced by PEG were observed under the microscope (Figure 1E). A total of 26 hybrid colonies were regenerated from five fusion experiments. No regenerated colonies were found in the control group. Sectors appearing in the protoplast fusion of distant hybrids are frequently observed when cultured in PDA, as shown in Figure 1H. This phenomenon is generally caused by the discordant division of heterokaryons from different sources, which separates one parent from unstable heterokaryons. Sixteen hybrids exhibited this particular phenomenon in PDA culture. The remaining 10 fusions are confirmed as hybrid strains, renamed R1 ~ R10. There were no single protocols developed from any of the parental protoplasts in this regeneration medium because protoplasts were inactivated. Only hybrid protoplasts could regenerate in a regeneration medium due to the complementation of the parental genome. This confirmed that 10 hybrids had dikaryotic hyphae, while the parent strain had monokaryotic hyphae, as observed under the fluorescence microscope (Figures 1B,F).

The colony morphology of hybrids was different from that of their parents. The colony morphology of *A. heimuer* cv. Bai Muer showed a whitish colony with linear and centrally radiating mycelia; *A. cornea* cv. Yu Muer produced a whitish colony with fluffy mycelia. The colony morphology of hybrids had the characteristics of both parents (Figures 1C,D,G).

### 3.4. Identification of hybrids

#### 3.4.1. Antagonistic reactions

**Figure 2:**
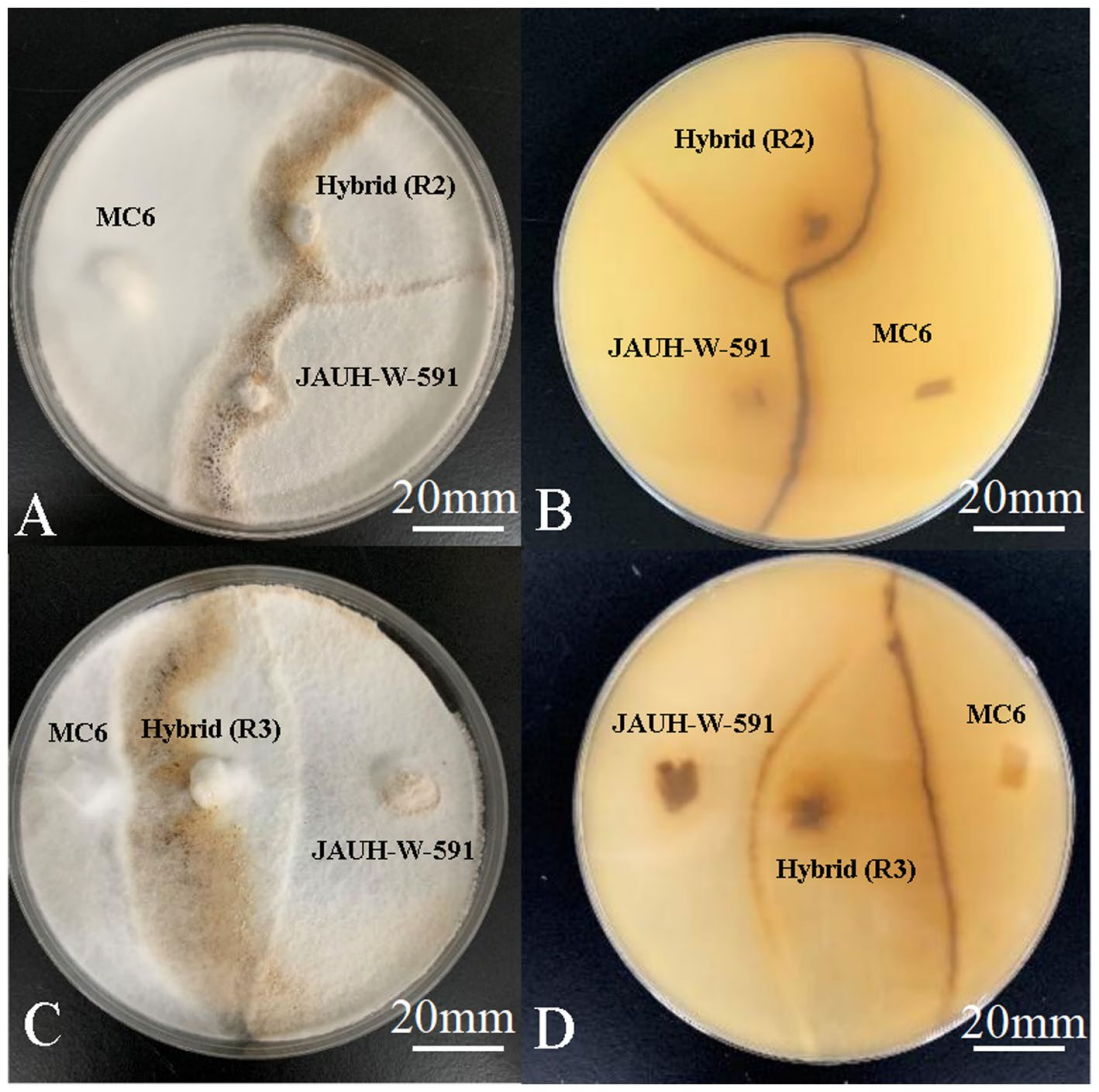
Antagonism of hybrids and parents. **(A-B)** The upper and reverse colony of JAUH-W-591, MC6, and R2; **(C-D)** the upper and reverse colony of JAUH-W-591, MC6, and R3.

The antagonistic reaction was a specific example of somatic incompatibility. The antagonist tests were conducted to confirm that the hybrids and parental strains had significant genetic differences. The antagonist test results showed that 10 hybrids and parental strains have a strong degree of antagonism resistance (Figure 2). This indicated that 10 strains generated through protoplast fusion are genetically different from the parental trains.

#### 3.4.2. Fruiting test of somatic hybrids

**Figure 3:**
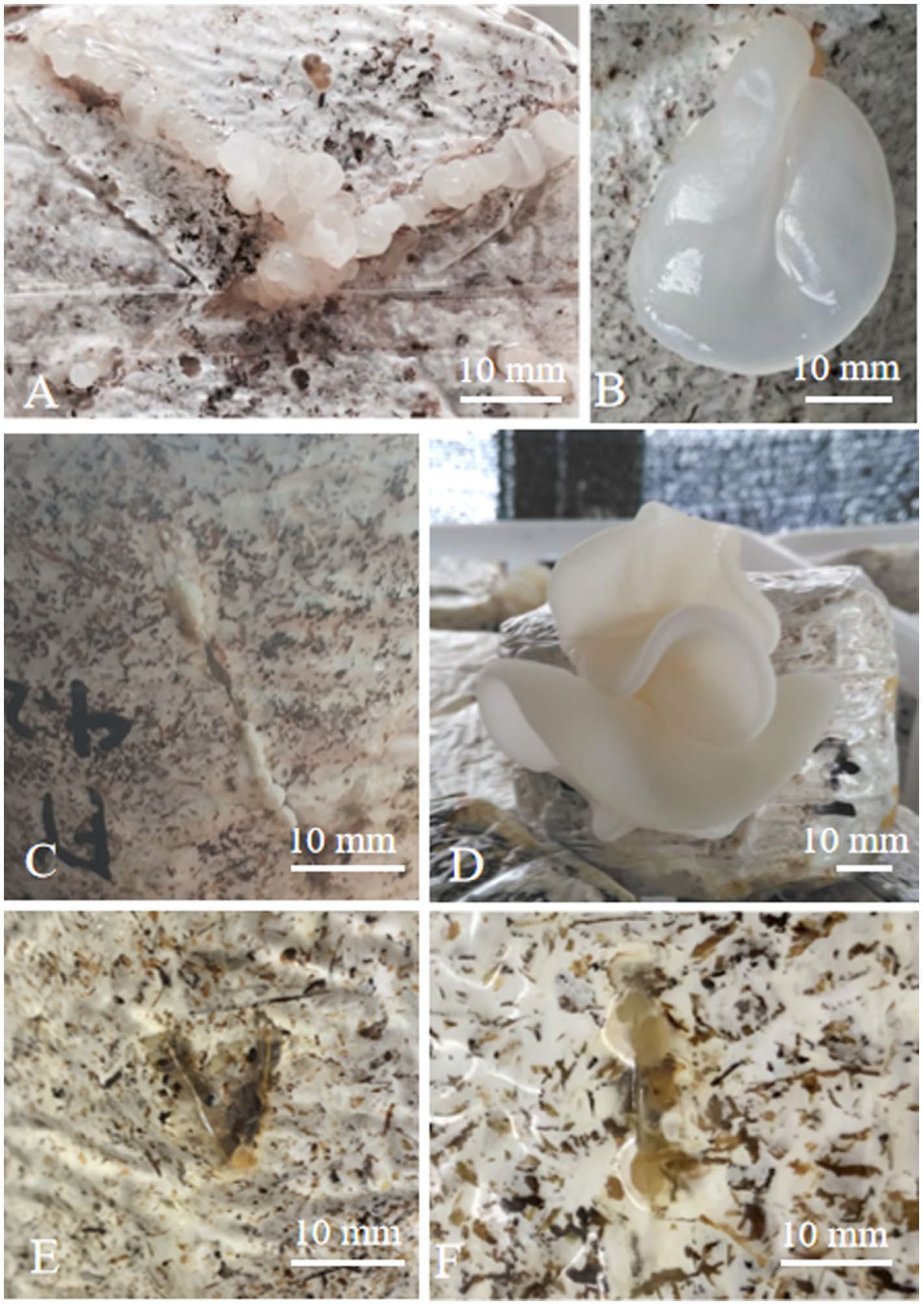
Morphology of fruiting bodies of hybrids **(A)** primordium of *A. heimuer* cv. Bai Muer; **(B)** fruiting body of *A. heimuer* cv. Bai Muer; **(C)** primordium of *A. cornea* cv. Yu Muer; **(D)** fruiting body of *A. cornea* cv. Yu Muer; and **(E,F)** primordium of hybrids.

In the cultivation study, hybrid mycelia grew thickly in a cultivation bag with sawdust as the main material. After 8 days of “V” pores being made, a yellowish-white primordium was observed in hybrid R2. Hybrid R4 required 11 days for primordial initiation, where parent *A. heimuer* cv. Bai Muer required 9 days and *A. cornea* cv. Yu Muer required 7 days. The results of lab-scale experiments indicated that all primordia of hybrids fail to differentiate even after 50 days. The fruit bodies of these parents and hybrids are shown in Figure 3.

#### 3.4.3. Inter-simple sequence repeat and start codon targeted analysis

**Figure 4:**
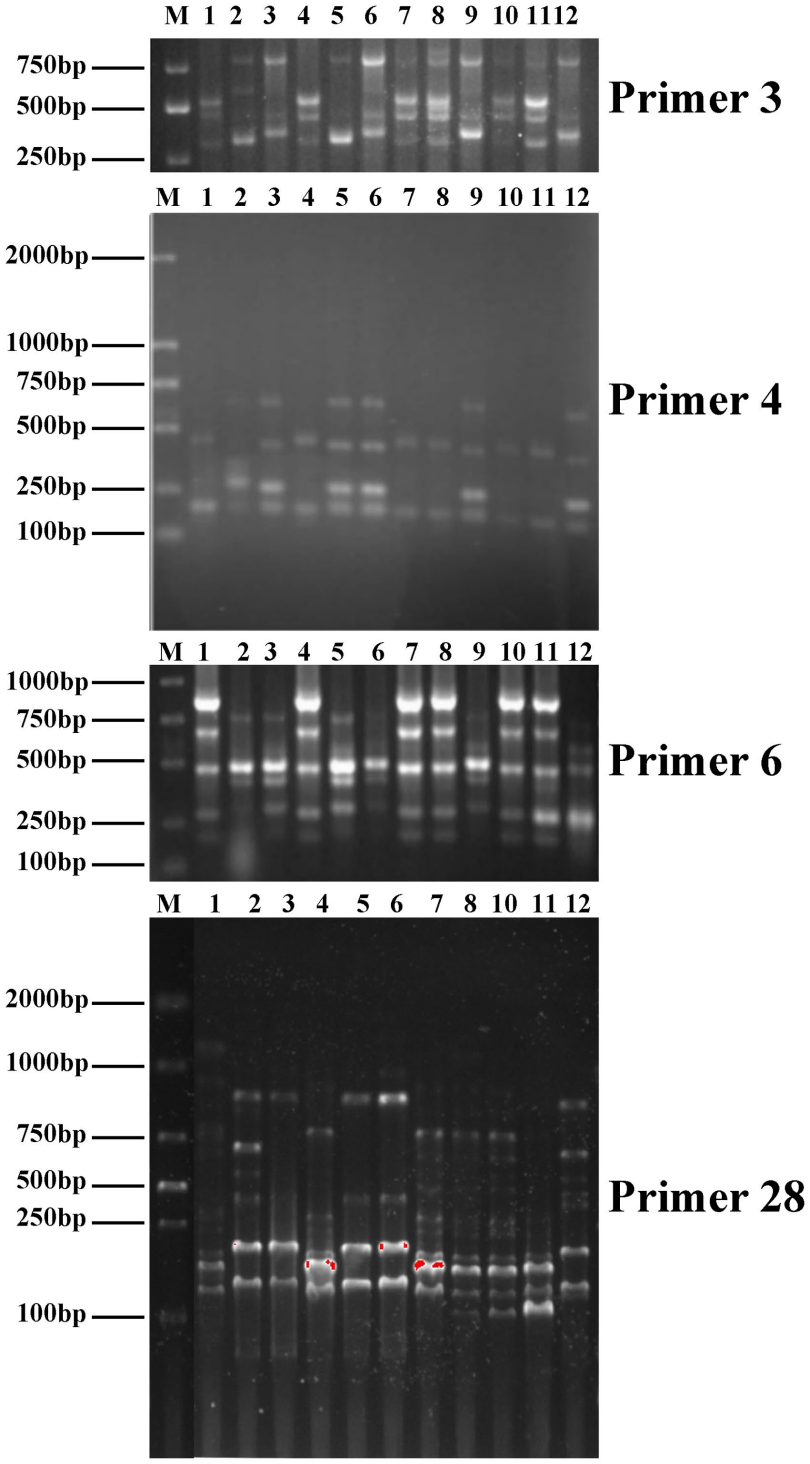
Inter-simple sequence repeat (ISSR) profiles of primers 3, 4, and 6 and start codon targeted (SCoT) profiles of primer 28 (1-R1, 2-R2, 3-R3, 4-R4, 5-R5, 6-R6, 7-R7, 8-R8, 9-R9, 10-R10, 11-*A. cornea* cv. Yu Muer, and 12-*A. heimuer* cv. Bai Muer).

Genetic variations among parental strains and 10 *Auricularia* hybrids were determined by using ISSR and SCoT markers. The PCR band profiles of the hybrids were compared with those of the parental strains. If ISSR and SCoT bands are present (or absent) in the possible fusion but not in the parents, they are considered distinct fragments. For example, the ISSR-3 primer amplified and generated new bands of approximately 600 bp in the R2. The ISSR-4 primer amplified a band of approximately 400 bp in *A. heimuer* cv. Bai Muer, but this band was not present in R2. Similarly, ISSR-6 could generate polymorphic bands in the R2, R3, R5, R6, and R9 strains. However, the band of approximately 250 bp appeared in both parent strains but was not present in the R2 and R6 strains. R3, R5, and R9 strains were similar to *A. cornea* cv. Yu Muer, but bands of approximately 700 bp and 850 bp appeared in *A. cornea* cv. Yu Muer were not present in the R3, R5, and R9 strains. SCoT-28 could generate polymorphic bands in all hybrids. This indicated that ISSR and SCoT were efficient in analyzing the genetic diversity of *Auricularia*. Meanwhile, SCoT markers have high polymorphism, a large amount of information, and a wide evaluation range, which are more suitable for genetic diversity research (Zhao et al., 2013). However, it has not been reported yet whether SCoT molecular markers have been applied to study the genetic diversity of *Auricularia*. In this study, we applied SCoT molecular markers to the genetic diversity of *Auricularia*, aiming to provide a reference for the construction of *Auricularia* molecular fingerprints and the evaluation of strains of *Auricularia*. The moderated genetic transformation was observed, as shown in Figure 4, and the hybrids obtained were confirmed to be heterokaryotic.

**Table 2:** Sequence and Annealing temperature of five start codon targeted (SCoT) primers.

Primer	Sequence	Annealing temperature (°C)
7	5’ CAACAATGGCTACCACGG 3’	51
8	5’ CAACAATGGCTACCACGT 3’	51
9	5’ CAACAATGGCTACCAGCA 3’	51
11	5’ AAGCAATGGCTACCACCA 3’	51
28	5’ CCATGGCTACCACCGCCA 3’	51

## 4. Discussion

Selecting true hybrids was an essential step in breeding, which can directly affect breeding efficiency. The PEG-induced double-inactivated protoplast has been widely applied in protoplast fusion in edible mushrooms (He et al., 2020). PEG is widely used to mediate cell–cell fusion in the production of somatic cell hybrids. PEG can cause changes in electron distribution on the cell surface in the presence of calcium ions. Then, fusion points and recesses form in the plasma membrane, constituting a bridge of protoplasts. Finally, intercellular channels were formed and gradually expanded until protoplast fusion was completed (Zhu et al., 2016). In this study, the protoplast-regenerated mononuclear strain of the parents was used as the starting strain of interspecific fusion, and different inactivation methods were used for marking inactivation to ensure that only fusion products regenerated into colonies. In the regeneration medium, neither the *A. heimuer* cv. Bai Muer protoplasts (due to heat inactivation) nor the *A. cornea* cv. Yu Muer protoplasts (due to UV inactivation) will grow. Hybrid protoplasts can grow due to the complementation of the parental genome or nuclear–cytoplasmic interactions (Mallick and Sikdar, 2014). The nuclear phase of the fused hybrid was observed as binucleate hypha under a fluorescence microscope, which showed that the genetic materials of both parents were complementarily repaired during the fusion process, and the heterokaryons were successfully obtained, which ruled out the possibility that the fused strain was the parent dikaryotic strain and monokaryon strain (Chiu et al., 1995).

This is the first time that interspecific protoplast fusion has been carried out among white varieties of *Auricularia* species. Yellowish-white primordia were obtained from two hybrids. The antagonistic line showed rejection between the fusion strain and its parents. The morphology of hybrids on the PDA medium had the characteristics of their parents. However, it may be due to the special mechanism of heterokaryon development after fusion or the change in environmental requirements of the fusion strain (Eyini et al., 2006); the primordia have not developed into a fruiting body, so it needs to be further domesticated and cultivated.

There are reports of the hybrids exhibiting novel nutrient and biochemical characteristics even though they resembled any of their parents molecularly (Loveleen and Kapoor, 2014; Mallick and Sikdar, 2016). Many different molecular markers, such as simple sequence repeats (SSRs), randomly amplified polymorphic DNA (RAPD), and sequence-characterized amplified region (SCAR), were used to find evidence of gene recombination (Yoo et al., 2002; Su et al., 2008; Mallick et al., 2017). Therefore, it is necessary to establish an accurate and rapid PCR-based diagnostic system for hybrid strains of white *Auricularia* hybrids. Moreover, the ISSR and SCoT primers are suitable for *A. heimuer* cv. Bai Muer and *A. cornea* cv. Yu Muer that are screened to reveal high polymorphism, which helps distinguish individuals at the inter- and/or intra-species level.

Post-fusion incompatibility caused by heterokaryons is common in mushrooms and has been reported in several mushrooms (Peberdy and Fox, 2018). Separating one parent from unstable heteronuclear cells in PDA culture proves this point. This phenomenon of parental separation is caused by the disharmony of heterokaryotic nuclei in distant fusion. Although protoplast fusion can bypass the natural barriers of cytoplasmic fusion and achieve distant hybridization between different species, protoplast fusion cannot eliminate the hybridization barriers caused by post-fusion incompatibility during hybrid development. In our experiment, we observed that the primordia of hybrids failed to differentiate into fruiting bodies. How to maintain the stability of heterokaryons is a crucial problem during the development of distant hybrids (Kim et al., 1997). Regardless of the genetic mechanism, when two distant parents undergo protoplast fusion, the resulting hybrids can offer a range of benefits. These benefits include enhanced biological efficiency, increased fruiting body yield, higher polysaccharide content, enhanced enzyme production, and other improvements (Okamura et al., 2000; Khattab and Mohamed, 2012; Das et al., 2021). This method has been proven to be successfully used in the improvement of naturally incompatible strains (Chakraborty and Sikdar, 2010). In addition, protoplast fusion may result in interactions between nuclear and exonuclear genes, such as mitochondrial genes (Stasz and Harman, 1990; Harman and Stasz, 1991; Harman and Hayes, 1993). Fukuda has reported the successful mitochondrial DNA transmission in interspecific fusion protoplasts of *Pleurotus*, which increased the genetic variability of economically significant mushrooms (Fukuda et al., 2007). Because mitochondrial genomes may influence the phenotypic characteristics of edible mushrooms, this possibility is useful in mushroom breeding (Zhao and Chang, 1997).

In this study, the double-inactivated method, colony morphology, barrage reaction, ISSR, and SCoT strongly proved their hybrid nature. The somatic hybrids obtained through this study are not end products. Instead, the non-fruit body-generating somatic hybrid could serve as resource material for backcrossing with parents, and other further studies would give us insight into the basic genetics of Basidiomycetes mating-type genes, clamp formation, and mode of sexuality. Moreover, these hybrids could be used for further mushroom improvement programs.

## Data availability statement

The original contributions presented in the study are included in the article, further inquiries can be directed to the corresponding authors.

## Author contributions

KQ: Conceptualization, Data curation, Investigation, Methodology, Software, Writing – original draft, Writing – review & editing. ZQ: Conceptualization, Software, Writing – review & editing, Data curation, Formal analysis, Methodology, Supervision, Writing – original draft. AX: Supervision, Project administration, Investigation, Methodology, Writing – review & editing, Conceptualization. BZ: Supervision, Project administration, Investigation, Methodology, Validation, Writing – review & editing. XL: Supervision, Project administration, Validation, Investigation, Writing – review & editing, Funding acquisition, Methodology, Resources. YL: Methodology, Supervision, Validation, Writing – review & editing.
